# The complete chloroplast genome sequence of *Xerophyta spekei* (Velloziaceae)

**DOI:** 10.1080/23802359.2019.1698365

**Published:** 2019-12-13

**Authors:** Vincent Okelo Wanga, Xiang Dong, Millicent Akinyi Oulo, Jacinta Ndunge Munyao, Elijah Mbandi Mkala, Paul Kirika, Robert Wahiti Gituru, Guang-Wan Hu

**Affiliations:** a CAS Key Laboratory of Plant Germplasm Enhancement and Specialty Agriculture, Wuhan Botanical Garden, Chinese Academy of Sciences, Wuhan, Hubei, China;; b University of Chinese Academy of Sciences, Beijing, China;; c Sino-Africa Joint Research Center, Chinese Academy of Sciences, Wuhan, Hubei, China;; d East African Herbarium, National Museums of Kenya, Nairobi, Kenya;; e Botany Department, Jomo Kenyatta University of Agriculture and Technology, Nairobi, Kenya

**Keywords:** *Xerophyta spekei*, Velloziaceae, chloroplast, genome sequence, phylogeny

## Abstract

The complete chloroplast genome sequence of *Xerophyta spekei* Baker was reported in this study. The complete chloroplast genome showed a stereotypical quadripartite structure as observed in other angiosperms with a length of 155,235 bp and divided into four parts; a pair of IRs (27,109 bp) which is separated by a small single copy (SSC) region (17,388 bp) and a large single copy (LSC) region (83,629bp). The chloroplast genome had 132 genes, including 85 protein-coding genes, 38 tRNA genes, and 8 rRNA genes. Seven protein-coding genes were identified to have RNA editing.

*Xerophyta spekei* Baker is a much branched shrub that grows 2–5 m tall in rocky outcrops at elevations from 300–1900 m (Beentje 1994; Pócs and Luke 2007). It is distributed in Kenya, Tanzania, Zambia, Zimbabwe, and possibly also in Ethiopia (Beentje 1994; Behnke et al. 2013). *Xerophyta* spp. with the exception of *Xerophyta elegans* are known to be poikilochlorophyllous, i.e. they lose their chlorophyll during desiccation (Mello-Silva et al. 2011; Behnke et al. 2013; Farrant et al. 2015). They are better adapted to xeric environments with specific substrates for their growth and development hence enhancing their endemism (Behnke et al. 2013; Farrant et al. 2015). *Xerophyta spekei* is a useful traditional medicinal plant whereby the leaf is used in case of stiffness of neck or other body parts; a piece of cloth is put on the aching part, and the area is rubbed with the warmed leaf and also the ashes are used to treat burns and diabetes (Kareru, Kenji, and Gachanja 2007; Kisangau and Herrmann 2007). Additionally, the stem is pounded and made into very strong brooms and paintbrushes and unspecified plant parts are used for cleaning metal pans and utensils (Beentje 1994).

Leaf samples were collected from Kibwezi, Chyulu National Park (Chyulu Base II) (02″44′18.94S, 037″56′41.04E), Kenya. The sample includes fresh and young photosynthetic leaves of *X. spekei* (SAJIT-006336). The collected samples were kept in silica gel and stored at −80 °C in Wuhan Botanical Garden (HIB) until chloroplast DNA extraction. The total genomic DNA was extracted using a modified cetyltrimethylammonium bromide (CTAB) method (Doyle 1991) and sequenced using the Illumina platform at Novogene Company (Beijing, China). After filtering the low-quality data and adaptors, the obtained clean data were assembled by GetOrganelle-1.6.2 (Jin et al. 2018), and then manually corrected. Finally, we used the geneious to find the IR region and annotated using PGA. The complete chloroplast genome of *Xerophyta spekei* showed four-part annular structures similar to most land plants. The length of the Complete Cp genome of *X. spekei* was 155,235 bp with a quadripartite structure and contained a pair of IRs (27,109 bp) which is separated by a small single copy (SSC) region (17,388 bp) and a large single copy (LSC) region (83,629 bp). The *X. spekei* Cp genome had 132 genes, including 85 protein-coding genes, 38 tRNA genes, and 8 rRNA genes. The overall GC content of *Xerophyta spekei* was 37.6%, with LSC, SSC and IR regions having 35.5, 31.8, and 42.5%, respectively.

Phylogenetic analysis was performed using whole chloroplast genome for the families; Cyclanthaceae, Pandanaceae, Stemonaceae, Triuridaceae found in the order pandanales; with one species from family Dioscoreaceae used as an out-group based on a previous study (Mennes et al. 2013), and an additional species from a closely related family Amaryllidaceae. All the nine taxa were aligned by MAFFT and the phylogenetic relationships were reconstructed by means of maximum-likelihood (ML) performed by IQ-Tree that is integrated in Phylosuite (Zhang et al. 2018) a GUI-based software written in Python 3.6.7. The phylogenetic tree was divided into two groups with *X. spekei* showing a closer relationship to species in the families’ Triuridaceae and Amaryllidaceae (Figure 1).

**Figure 1.**
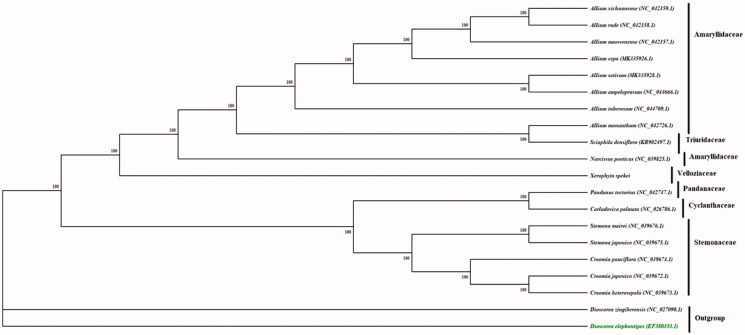
Phylogenetic tree based on maximum parsimony analysis of *X. spekei* with related species. The numbers above the branches are the bootstrap statistics values from 1000 replications.
